# Endocardial border delineation capability of a novel multimodal polymer-shelled contrast agent

**DOI:** 10.1186/1476-7120-12-24

**Published:** 2014-07-03

**Authors:** Malin K Larsson, Matilda Larsson, Greg Nowak, Gaio Paradossi, Lars-Åke Brodin, Birgitta Janerot Sjöberg, Kenneth Caidahl, Anna Bjällmark

**Affiliations:** 1 Department of Medical Engineering, School of Technology and Health, KTH Royal Institute of Technology, Alfred Nobels Allé 10, 141 52 Huddinge, Sweden; 2 Department of Molecular Medicine and Surgery, Karolinska Institutet, Solna, Sweden; 3 Department of Clinical Science, Intervention and Technology, Karolinska Institutet, Stockholm, Sweden; 4 Department of Chemical Sciences and Technologies, Università di Roma Tor Vergata, Rome, Italy; 5 Department of Clinical Physiology, Karolinska University Hospital, Stockholm, Sweden

**Keywords:** Contrast agent, Echocardiography, Endocardial border delineation, Microbubbles, Polyvinyl alcohol, Porcine, Ultrasound

## Abstract

**Background:**

A novel polymer-shelled contrast agent (CA) with multimodal and target-specific potential was developed recently. To determine its ultrasonic diagnostic features, we evaluated the endocardial border delineation as visualized in a porcine model and the concomitant effect on physiological variables.

**Methods:**

Three doses of the novel polymer-shelled CA (1.5 ml, 3 ml, and 5 ml [5 × 10^8^ microbubbles (MBs)/ml]) and the commercially available CA SonoVue (1.5 ml [2–5 × 10^8^ MBs/ml]) were used. Visual evaluations of ultrasound images of the left ventricle were independently performed by three observers who graded each segment in a 6-segment model as either 0 = not visible, 1 = weakly visible, or 2 = visible. Moreover, the duration of clinically useful contrast enhancement and the left ventricular opacification were determined. During anesthesia, oxygen saturation, heart rate, and arterial pressure were sampled every minute and the effect of injection of CA on these physiological variables was evaluated.

**Results:**

The highest dose of the polymer-shelled CA gave results comparable to SonoVue. Thus, no significant difference in the overall segment score distribution (2-47-95 vs. 1-39-104), time for clinically sufficient contrast enhancement (20–40 s for both) and left ventricular overall opacification was found. In contrast, when comparing the endocardial border delineation capacity for different regions SonoVue showed significantly higher segment scores for base and mid, except for the mid region when injecting 1.5 ml of the polymer-shelled CA. Neither high nor low doses of the polymer-shelled CA significantly affected the investigated physiological variables.

**Conclusions:**

This study demonstrated that the novel polymer-shelled CA can be used in contrast-enhanced diagnostic imaging without influence on major physiological variables.

## Background

Intravenously injectable contrast agents (CA) consisting of gas-filled microbubbles (MBs) with a mean diameter of 2–5 μm can be used for improved image quality during ultrasound examinations, which results in diagnostic benefits. An ideal ultrasound CA must be biocompatible, stable during image acquisition, and circulate without causing obstructions or negative physiological effects. In addition, it should have the ability to increase backscattering efficiency when exposed to an acoustic field. In 1968, the first contrast-enhanced echocardiography examination was described [1]. At that time, fragile free-air MBs with a short lifetime of a few seconds were used. Since then, more stable CAs have been developed by encapsulating a low-solubility gas within a stabilizing shell [2]. In parallel, the development of specific ultrasound contrast pulse sequences, which use the nonlinear response generated by the MBs when exposed to acoustic pressure, has further improved the possibility of successfully using these CAs during ultrasound examinations [3,4].

Although commercially available ultrasound CAs are relatively stable and used in various diagnostic procedures, there is still a need for improvement and extended applicability. One such example is targeted imaging, which would enable the visualization of specific areas, e.g., inflammatory tissue. Local deposition of drugs might also be performed using a target-specific and drug-loaded CA. Moreover, increased diagnostic efficiency and accuracy can be achieved when using a multimodal CA, which supports hybrid imaging by applying two or more modalities to produce anatomical and functional information simultaneously.

A novel polymer-shelled CA with high mechanical and chemical stability was developed recently [5]. Further to its ultrasound properties, it also supports targeted and multimodal imaging (ultrasound, magnetic resonance imaging, and emission imaging) because the chemical versatility of its shell surface enables attachment of different substances such as antibodies, superparamagnetic iron oxide nanoparticles (SPIONs) and technetium [6-9]. Even though these new possibilities have the potential to lead to new methodologies and approaches for noninvasive diagnosis, it is important that the diagnostic features in contrast-enhanced ultrasound are preserved. The aim of this study was therefore to investigate the left ventricular endocardial border delineation capability in ultrasound images of a porcine model when using the polymer-shelled CA. In addition, the effect of CA injection on physiological variables was studied.

## Methods

To assess the endocardial border delineation capability of the unmodified air filled polymer-shelled CA, a comparative study, including a commercially available lipid monolayer membrane-shelled CA containing sulfur hexafluoride (SonoVue, Bracco Imaging, Milan, Italy), was performed using a porcine model. Visual interpretations were performed by independent observers who were blinded to CA type and dose. Additionally, semiautomatic segmentation was applied to provide information regarding the uniformity of left ventricular opacification.

### Animals

The study protocol was approved by the local Ethics Committee for Animal Experiments in Stockholm, Sweden. Eight crossbred (Swedish Landrace, Yorkshire, or Hampshire) littermate female open-chest pigs with a body weight of 34.6 ± 3.1 kg were included in the study. The pigs were fasted overnight before surgery with free access to water. Anesthesia was induced with an intramuscular injection of fentanyl and atracurium before endotracheal intubation. Subsequently anesthesia was maintained with 1–2% isoflurane ventilation and was augmented with fentanyl and atracurium intravenous injection when required. Animals were continuously monitored with ECG, pulse oximetry, and invasive arterial blood pressure. Liometacen (Promedica, Parma, Italy) was intravenously injected (10 mg/kg, 5 mg/kg/h) to prevent pulmonary and systemic hypertension [10].

### Contrast agents

The novel polymer-shelled contrast agent is produced by cross-linking of synthesized polyvinyl alcohol at the air-water interface during high-shear stirring with an Ultra Turrax (IKA, Staufen, Germany) stirrer at 8000 rpm [5], resulting in air-filled MBs with a mean diameter of 4.6 μm ± 0.4 μm and a shell thickness of 0.4 μm [11]. In each pig, three bolus doses (1.5 ml, 3 ml, and 5 ml) of the polymer-shelled CA [5 × 10^8^ MBs/ml] and 1.5 ml of SonoVue [2–5 × 10^8^ MBs/ml] were manually injected through a central venous catheter placed in the external jugular vein, at a speed of approximately 0.75 ml/s. In addition, a 4 ml flush of 0.9% saline followed each CA injection. The subsequent CA injection started after about 10 minutes when no contrast enhancement could be observed in the left ventricle. SonoVue and the different doses of the polymer-shelled CA were injected in a randomized order in each animal.

### Image acquisition

For ultrasound imaging, a commercially available clinical ultrasound machine was used (iE33; Philips Healthcare, Best, The Netherlands). Ultrasound-imaging settings such as contrast sequences, frequency, mechanical index (MI), focus depth, and gain were individually optimized for each CA according to in vitro [8] and in vivo pilot studies (see Table 1). ECG-triggered ultrasound images from the apical two-chamber view were acquired by an experienced echocardiographer at end-systole. The image acquisition was stopped when no or very low contrast enhancement was visually observed in the left ventricle.

**Table 1:** Characteristics of the ultrasound settings used for the two contrast agents

**CA**	**Contrast sequence**	**Frequency (MHz)**	**MI**
Polymer-shelled CA	Power pulse inversion	1.6/3.2	0.89–0.93
SonoVue	Power modulation	1.6/3.2	0.39

CA = contrast agent, MI = mechanical index.

### Visual evaluation

**Figure 1:**
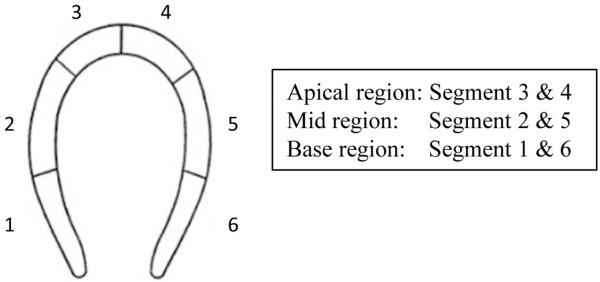
**The 6-segment model used for image analysis.** The endocardial border delineation for each segment was graded as 0 = not visible, 1 = weakly visible, or 2 = visible.

For each injection, the obtained image series were divided in time intervals of 20 seconds, starting when inflow of CA into the left ventricle was observed. From each time interval, the image with the highest and most homogeneous contrast enhancement was selected for further analysis using a Q-Lab workstation (Philips Healthcare, Best, The Netherlands). Among these images, experienced observers (n = 3), who were blinded to CA type and dose, visually selected the image with best potential for endocardial border delineation. This was done individually by each observer. The endocardial border delineation of the selected image was evaluated by the observers using a 6-segment model (see Figure 1). Every segment was graded as 0 = not visible, 1 = weakly visible, or 2 = visible. Furthermore, the duration of clinically useful contrast enhancement was evaluated by the three observers visually (while still blinded to dose and CA type), and the mean time was calculated for each dose and injection.

### Semiautomatic delineation

Segmentation software based on level-set developed in MATLAB (MathWorks, Natick, MA, USA) [12,13] was used to evaluate semiautomatic border definition in the ultrasound images. Because there is no clear difference in signal intensity between a contrast-enhanced atrium and a contrast-enhanced ventricle, the segmentation software was modified for the current application by allowing manual definition of the atrioventricular plane*.* Furthermore, the number of iterations (2500) and Caselles algorithm [13] were selected by initial testing to fit the current application.

The same set of images as selected by the observers for the visual evaluation was employed in the semiautomatic delineation. In these images, an initial elliptical region (with an area of approximately a quarter of the total area of the left ventricle) was manually placed in the center of the left ventricle and thereafter the automated delineation was initiated. In addition, the experienced observers performed manual delineation of the endocardial border by pointing out 20 border points in the image followed by spline interpolation to delineate the reference border. The overlap between the reference border and semiautomatic delineation was evaluated by calculating the Dice value (D) as:

$$D\;\left(A,\;B\right)\;=\;2\;|A\;\cap \;B|/\;\left(|A|+|B|\right),$$

where A was the set from the semiautomated delineation and B the set from the reference border. A higher Dice value corresponded to a better delineation match between the reference and semiautomatic delineation. A Dice value of 1 corresponded to an ideal match.

### Physiological data

During anesthesia, oxygen saturation (SaO_2_), heart rate, and arterial pressure were sampled every minute. After the final injection dose, data was exported and analyzed offline.

### Statistical analysis

The differences in overall segment score (n = 144) and in segment score for different wall-regions (apical, mid, and base, each n = 48) between SonoVue and the three doses of the polymer-shelled CA were evaluated using a Wilcoxon signed-rank test. The effect of injection of CA on the physiological variables was evaluated by comparing the relative difference between 3 minutes before and after injection of each dose using a paired two-sided Student *t* test (95% confidence interval). Furthermore, a McNemar test was used to compare the Dice value distribution of the polymer-shelled CA and SonoVue for three different Dice value intervals (<0.8; 0.8–0.9; >0.9).

## Results

### Visual evaluation

The segment score distribution for each dose and CA is presented in Table 2. When considering all segments within the left ventricle, there was no significant difference in segment score between the 5 ml injection of the polymer-shelled CA and SonoVue, indicating no significant difference in the overall capability of the endocardial border delineation for the two CAs at these doses. By comparison with SonoVue, significantly lower segment scores were obtained for the polymer-shelled CA at doses lower than 5 ml.

**Table 2:** The segment visibility score distribution for each dose and CA injected

**CA (injected dose)**	**All segments**			**Apical**			**Mid**			**Base**		
	**(n = 144)**			**(n = 48)**			**(n = 48)**			**(n = 48)**		
	**0**	**1**	**2**	**0**	**1**	**2**	**0**	**1**	**2**	**0**	**1**	**2**
SonoVue (1.5 ml)	1	39	104	1	33	14	0	0	48	0	6	42
Polymer-shelled CA (1.5 ml)	4	49	91	1	26	21	0	3	45	3	20	25
	(*P* < 0.05)			(NS)			(NS)			(*P* < 0.001)		
Polymer-shelled CA (3 ml)	12	48	84	3	27	18	3	4	41	6	17	25
	(*P* < 0.001)			(NS)			(*P* < 0.05)			(*P* < 0.001)		
Polymer-shelled CA (5 ml)	2	47	95	2	22	24	0	4	44	0	21	27
	(NS)			(NS)			(*P* < 0.05)			(*P* < 0.05)		

A significant difference in segment scores between SonoVue and the polymer-shelled CA is indicated by its *P*-value and a nonsignificant difference with NS (paired by segment and pig using a Wilcoxon signed-rank test).

CA = contrast agent (0 = not, 1 = weakly, 2 = visible).

**Figure 2:**
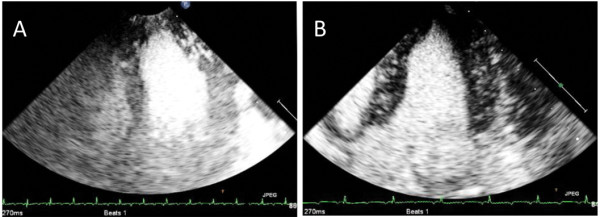
**Ultrasound contrast images of the porcine left ventricle.** Injection of 1.5 ml of the polymer-shelled CA **(A)** and 1.5 ml of SonoVue **(B)**.

When comparing segment scores for different wall-regions (apical, mid, and base) within the left ventricle, no significant difference in segment score was observed for SonoVue or any of the polymer-shelled CA injections for the apical region (Table 2). For the other two regions (mid and base), SonoVue showed significantly better performance than all three doses of the polymer-shelled CA, with one exception in the mid region when injecting 1.5 ml. Examples of ultrasound images obtained during the examination for both CAs are shown in Figure 2.

According to the results from the observers, the longest detectable time of clinical useful contrast enhancement was obtained when injecting 5 ml of the polymer-shelled CA and 1.5 ml SonoVue (see Table 3). The clinically useful time periods of contrast enhancement for these two injections were in the range of 20–40 seconds, while the first time interval (0–20 seconds) was observed for the other two injections.

**Table 3:** Time period of clinically useful contrast enhancement after CA injection

**CA (injected dose)**	**Clinically useful time period**
SonoVue (1.5 ml)	20–40 s
Polymer-shelled CA (1.5 ml)	0–20 s
Polymer-shelled CA (3 ml)	0–20 s
Polymer-shelled CA (5 ml)	20–40 s

CA = contrast agent.

### Semiautomatic delineation

**Figure 3:**
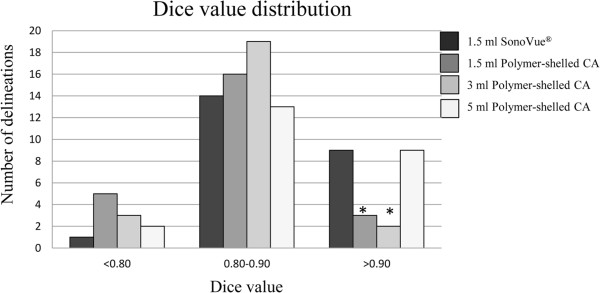
**Dice value distribution.** The number of delineations (3 observers x 8 pigs) for each dose (1.5 ml SonoVue; 1.5 ml, 3 ml, or 5 ml polymer-shelled CA) distributed over Dice value ranges of <0.80, 0.80–0.90, and >0.90. A higher Dice value corresponds to a better match with the reference delineation. *Significant difference (*P* < 0.05) in Dice value distribution compared with SonoVue.

**Figure 4:**
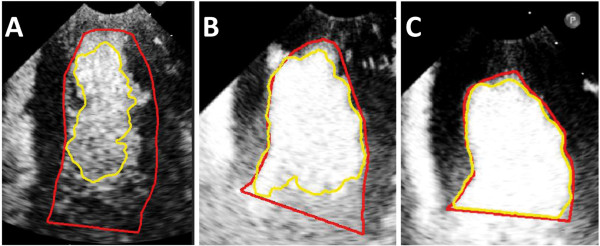
**Illustration of the results obtained from the semiautomatic delineation.** The red line represents the reference delineation and the yellow line represents the semiautomatic delineation. **(A)** 1.5 ml polymer-shelled CA, Dice value = 0.61; **(B)** 3 ml polymer-shelled CA, Dice value = 0.82; **(C)** 5 ml polymer-shelled CA, Dice value = 0.95.

Figure 3 shows the Dice value distribution (<0.8; 0.8–0.9; >0.9) for all doses (n = 24). As can be seen, similar distribution of Dice values were obtained for 1.5 ml SonoVue and the 5 ml injection of the polymer-shelled CA. By contrast with the lower doses (1.5 ml and 3 ml) of the polymer-shelled CA, these two doses had a distribution shifted towards the highest Dice value interval (>0.90), which indicated a better match with the reference delineation. The McNemar test further indicated that there was a significant difference in distribution for the highest Dice value between the lower doses and SonoVue. Figure 4 illustrates the difference in homogeneity of the left ventricular opacification when injecting 1.5 ml (Figure 4A), 3 ml (Figure 4B), and 5 ml (Figure 4C) of the polymer-shelled CA.

### Physiological data

Table 4 presents the mean baseline values for the investigated physiological variables obtained before CA injection. No significant change in SaO_2_, heart rate, or mean arterial pressure for any dose of the polymer-shelled CA was observed. Table 5 shows the mean percentage differences before and after CA injection between the physiological variables.

**Table 4:** The mean value for the physiological variables obtained before CA injection

**SaO**_**2**_	**Heart rate**	**Mean arterial pressure**
**(%)**	**(bpm)**	**(mmHg)**
98 ± 1.5	93 ± 14	78 ± 13

CA = contrast agent, SaO_2_ = oxygen saturation.

**Table 5:** Physiological parameters before and after CA injection

**CA (injected dose)**	**SaO**_**2**_	**Heart rate**	**Mean arterial pressure**
	**(%)**	**(bpm)**	**(mmHg)**
SonoVue (1.5 ml)	0% ± 0	0% ± 1	5% ± 9
Polymer-shelled CA (1.5 ml)	0% ± 0	0% ± 2	-2% ± 2
Polymer-shelled CA (3 ml)	0% ± 0	-4% ± 8	-1% ± 4
Polymer-shelled CA (5 ml)	0% ± 0	-2% ± 3	2% ± 3

CA = contrast agent, SaO_2_ = oxygen saturation. Mean percentage difference ± standard deviation.

## Discussion

The use of CA during ultrasound examinations improves endocardial border delineation in patients with suboptimal ultrasound images [14-16]. This enables a more accurate assessment of left ventricular wall motion, wall-thickening abnormalities, volume measurements, and calculation of ejection fraction. These variables are crucial factors for characterization and prognosis of cardiac diseases such as myocardial ischemia and myocardial infarction [17,18].

This study showed that the performance for delineation of the endocardial border of 5 ml of the novel polymer-shelled CA was comparable to 1.5 ml of the commercially available CA SonoVue. This performance included similar values of overall segment scores and time periods for clinically sufficient contrast enhancement as rated by the observers (see Tables 2 and 3). In addition, the results from the semiautomatic segmentation software were in agreement with this, showing equal distribution of Dice values for SonoVue and the 5 ml injection of the polymer-shelled CA, which indicated a uniform left ventricular opacification for the two CAs. Nevertheless, this study also implies that the polymer-shelled CA does not have the same backscattering efficiency as SonoVue because a larger volume is needed to obtain similar contrast enhancement. The difference in shell thickness between the two types of CAs, where the polymer-shelled CA has a thicker and stiffer shell than SonoVue [11,19], is suggested to be the cause of this difference. A thicker shell is less flexible and has considerable damping, which causes a reduction in MB radius oscillation, and therefore a decrease in the nonlinear response [20]. Despite this, the advantage of the polymer-shelled CA is that its shell can be decorated by different substances to enable multimodal and targeting imaging. Improvements in designing ultrasound contrast pulse sequences optimized for the polymer-shelled CA could increase its diagnostic capabilities. On the other hand, results from this study show that a high concentration of the polymer-shelled CA is applicable from a purely echocardiographic point-of-view when using commercially available contrast sequences.

This study employed an unmodified polymer-shelled CA to obtain an indication of the approximate outcome in standard contrast-enhanced echocardiography. Attachment of ligands or incorporation of therapeutic gases or drugs may alter our ability to visualize the CA depending on the properties of the incorporated substance. This has been observed in both single-element set-ups [21,22] and in phantom studies using clinical ultrasound systems [8], where the latter showed that the introduction of SPIONs to the polymer-shelled CA increases backscattering efficiency when applying the same image-acquisition settings as in the present study. Even though unmodified MBs were used in the present study, the results give an indication of what can be expected when performing similar examinations with modified MBs.

A possible advantage of the polymer-shelled CA compared with SonoVue in the context of endocardial border delineation is the fact that the polymer-shelled CA tolerates higher pressure amplitudes before the MBs rupture. When performing contrast-enhanced imaging with thin-shelled CA such as SonoVue, there is a trade-off between visualization of deep structures of the heart, where high-pressure amplitudes are optimal, and visualization of near-field regions, where low-pressure amplitudes are optimal, because high-pressure amplitudes rupture the MBs in the near-field regions. Thus, the polymer-shelled CA might be expected to allow better visualization of both the apical and the basal regions because higher MI was used for the polymer-shelled CA (MI = 0.89–0.93) compared with SonoVue (MI = 0.39). However, this was not seen in the present study. One possible explanation is that the ultrasound beam was attenuated when high-pressure amplitudes in combination with a large dose of the polymer-shelled CA were used, which resulted in decreased imaging depth [23]. Another possible explanation could be that trigged imaging was used. When employing trigged imaging for SonoVue, MI can be higher than during continuous imaging which in turn optimizes the visualization of the base.

The scoring results from the three observers were unanimous. All observers indicated the same ranking of the different CA doses, where SonoVue and the 3 ml injection of the polymer-shelled CA received the highest and the lowest segment scores by each observer, respectively. However, the segment scores for each CA dose differed between the observers. In general, the same observer reported the highest segment scores, while the lowest segment scores were observed by another observer. This consistency among the observers indicates that the model used to evaluate the CAs is robust. Because the semiautomatic delineation was evaluated against the manual reference, no objective analysis was obtained. All observers had extensive experience analyzing cardiac contrast-enhanced ultrasound images and their reference could therefore be used as the adequate delineation of the left ventricle. A limitation of the analysis is that intermittent imaging was used, which implied that the observer could not step back and forward in the image sequence when some parts of the endocardium were not fully visible because of, e.g., attenuation effects. This might have produced lower segment scores than if continuous imaging had been employed. Moreover, the ability to evaluate regional wall motion or to fully optimize left ventricular volume measurements was not possible with these settings. Nevertheless, by employing intermittent imaging in this study, visual evaluation and semiautomatic delineation could be performed under equivalent conditions. The ability of the novel polymer-shelled CA to detect cardiac abnormalities is a secondary step and will be evaluated in future work.

The performance of semiautomatic delineation provides information about the ability to delineate the endocardial border and the uniformity of left ventricular opacification, which is seen clearly in Figure 4A-B. A homogenous and persistent left ventricular opacification is of great importance in contrast-enhanced echocardiography because previous studies have shown that a complete left ventricular opacification results in up to 95% improvement in endocardial border resolution [24].

Even though a relatively large dose of the CAs were injected in each pig, no significant variation in the physiological variables was seen during the experiment, which indicated a stable anesthesia procedure and no untoward hemodynamic effect of CA injections. On the other hand, a rather large CA dose could increase the risk of CA saturation after repeated injections. By randomizing the order of the CA injections in each pig, this risk was minimized. Furthermore, recovery periods between injections were also present. It was not possible to evaluate whether there was a difference when low doses of CA were distributed before or after high doses because there were relatively few injections. Moreover, minor contrast peaks during periods between the injections were not observed, which indicated the absence of CAs in the circulation.

## Conclusions

This study demonstrated that the novel polymer-shelled CA can be useful in contrast-enhanced diagnostic imaging for endocardial border delineation. The ability of the novel polymer-shelled CA to delineate endocardial border was comparable to SonoVue when injecting a high dose. This was shown by similar distribution for overall segment scores from visual observations, time for clinically sufficient contrast enhancement, and the same ability for semiautomatic delineation of the left ventricle. In addition, injections of the polymer-shelled CA did not significantly affect SaO_2_, heart rate, or arterial pressure. To evaluate its diagnostic capacity further, studies focused on perfusion imaging, multimodal imaging, and targeted imaging are warranted.

## Competing interests

The authors declare that they have no competing interests.

## Authors’ contributions

MKL, ML, LÅB, AB participated in the initiation and design of the study. MKL and GP produced the polymer-shelled CA used in the study. ML, ML, GN, KC and AB participated in data collection and analysis of the results. All authors read and approved the final manuscript.

## References

[B1] Gramiak R Shah PM Echocardiography of the aortic root Invest Radiol 1968 3 356 366 5688346 10.1097/00004424-196809000-00011

[B2] Schneider M Characteristics of SonoVuetrade mark Echocardiography 1999 16 743 746 11175217 10.1111/j.1540-8175.1999.tb00144.x

[B3] Averkiou M Powers J Skyba D Bruce M Jensen S Ultrasound contrast imaging research Ultrasound Q 2003 19 27 37 12970614 10.1097/00013644-200303000-00004

[B4] Kaufmann BA Wei K Lindner JR Contrast echocardiography Curr Probl Cardiol 2007 32 51 96 17208647 10.1016/j.cpcardiol.2006.10.004

[B5] Cavalieri F El Hamassi A Chiessi E Paradossi G Stable polymeric microballoons as multifunctional device for biomedical uses: synthesis and characterization Langmuir 2005 21 8758 8764 16142958 10.1021/la050287j

[B6] Brismar TB Grishenkov D Gustafsson B Harmark J Barrefelt A Kothapalli SV Margheritelli S Oddo L Caidahl K Hebert H Paradossi G Magnetite nanoparticles can be coupled to microbubbles to support multimodal imaging Biomacromolecules 2012 13 1390 1399 22458325 10.1021/bm300099f

[B7] Barrefelt AA Brismar TB Egri G Aspelin P Olsson A Oddo L Margheritelli S Caidahl K Paradossi G Dahne L Axelsson R Hassan M Multimodality imaging using SPECT/CT and MRI and ligand functionalized 99mTc-labeled magnetic microbubbles EJNMMI Res 2013 3 12 23442550 10.1186/2191-219X-3-12 PMC3599195

[B8] Larsson M Larsson M Oddo L Margheritelli S Paradossi G Nowak J Brodin LA Caidahl K Bjallmark A Visualization of multimodal polymer-shelled contrast agents using ultrasound contrast sequences: an experimental study in a tissue mimicking flow phantom Cardiovasc Ultrasound 2013 11 33 23987142 10.1186/1476-7120-11-33 PMC3766157

[B9] Villa R Cerroni B Vigano L Margheritelli S Abolafio G Oddo L Paradossi G Zaffaroni N Targeted doxorubicin delivery by chitosan-galactosylated modified polymer microbubbles to hepatocarcinoma cells Colloids Surf B: Biointerfaces 2013 110 434 442 23759384 10.1016/j.colsurfb.2013.04.022

[B10] Ostensen J Hede R Myreng Y Ege T Holtz E Intravenous injection of Albunex microspheres causes thromboxane mediated pulmonary hypertension in pigs, but not in monkeys or rabbits Acta Physiol Scand 1992 144 307 315 1533987 10.1111/j.1748-1716.1992.tb09299.x

[B11] Cavalieri F FInelli I Tortora M Mozetic P Chiessi E Polizio F Brismar TB Paradossi G Polymer micobubbles as diagnostic and therapeutic gas delivery device Chem Mater 2008 20 3254 3258

[B12] Dietenbeck T Alessandrini M Friboulet D Bernard O Creaseg: a free software for the evaluation of image segmentation algorithms based on level-set 2010 Hong Kong, China: IEEE Image Processing (ICIP)

[B13] Caselles V Kimmel R Sapiro G Geodesic active contours Int J Comput Vis 1997 22 61 79

[B14] Kitzman DW Goldman ME Gillam LD Cohen JL Aurigemma GP Gottdiener JS Efficacy and safety of the novel ultrasound contrast agent perflutren (definity) in patients with suboptimal baseline left ventricular echocardiographic images Am J Cardiol 2000 86 669 674 10980221 10.1016/s0002-9149(00)01050-x

[B15] Nanda NC Wistran DC Karlsberg RP Hack TC Smith WB Foley DA Picard MH Cotter B Multicenter evaluation of SonoVue for improved endocardial border delineation Echocardiography 2002 19 27 36 11884252 10.1046/j.1540-8175.2002.00027.x

[B16] Senior R Andersson O Caidahl K Carlens P Herregods MC Jenni R Kenny A Melcher A Svedenhag J Vanoverschelde JL Wandt B Widgren BR Williams G Guerret P La Rosee K Agati L Bezante G Enhanced left ventricular endocardial border delineation with an intravenous injection of SonoVue, a new echocardiographic contrast agent: a European multicenter study Echocardiography 2000 17 705 711 11153016 10.1111/j.1540-8175.2000.tb01223.x

[B17] Goldstein S de Jong JW Changes in left ventricular wall dimensions during regional myocardial ischemia Am J Cardiol 1974 34 56 62 4835754 10.1016/0002-9149(74)90093-9

[B18] White HD Norris RM Brown MA Brandt PW Whitlock RM Wild CJ Left ventricular end-systolic volume as the major determinant of survival after recovery from myocardial infarction Circulation 1987 76 44 51 3594774 10.1161/01.cir.76.1.44

[B19] Hoff L Acoustic characterization of contrast agents for medical ultrasound imaging 2001 AA Dordrecht: Kluwer Academic Publisher

[B20] Leong-Poi H Song J Rim SJ Christiansen J Kaul S Lindner JR Influence of microbubble shell properties on ultrasound signal: implications for low-power perfusion imaging J Am Soc Echocardiogr 2002 15 1269 1276 12411916 10.1067/mje.2002.124516

[B21] Poehlmann M Grishenkov D Kothapalli SVVN Härmark J Philipp A Höller R Seuss M Paradossi G Fery A On the interplay of structural, mechanical and acoustic behaviour of multifunctional magnetic microbubbles Soft Matter 2014 10 214 226 24651844 10.1039/c3sm51560e

[B22] Grishenkov D Pecorari C Brismar TB Paradossi G Characterization of acoustic properties of PVA-shelled ultrasound contrast agents: linear properties (part I) Ultrasound Med Biol 2009 35 1127 1138 19427099 10.1016/j.ultrasmedbio.2009.02.002

[B23] Chen Q Zagzebski J Wilson T Stiles T Pressure-dependent attenuation in ultrasound contrast agents Ultrasound Med Biol 2002 28 1041 1051 12217440 10.1016/s0301-5629(02)00546-x

[B24] Porter TR Xie F Kricsfeld A Chiou A Dabestani A Improved endocardial border resolution during dobutamine stress echocardiography with intravenous sonicated dextrose albumin J Am Coll Cardiol 1994 23 1440 1443 8176104 10.1016/0735-1097(94)90389-1

